# Transcriptome Analysis of Needle and Root of *Pinus Massoniana* in Response to Continuous Drought Stress

**DOI:** 10.3390/plants10040769

**Published:** 2021-04-14

**Authors:** Feng Xiao, Yang Zhao, Xiu-Rong Wang, Qiao Liu, Jie Ran

**Affiliations:** 1 College of Forestry, Guizhou University, Guiyang 550025, China; maplexiao594@gmail.com (F.X.); wxr7211@126.com (X.-R.W.); liuqiao20200101@163.com (Q.L.); xuey520@126.com (J.R.); 2 Institute for Forest Resources & Environment of Guizhou, Guizhou University, Guiyang 550025, China; 3 Key Laboratory of Forest Cultivation in Plateau Mountain of Guizhou Province, Guizhou University, Guiyang 550025, China; 4 Key Laboratory of Plant Resource Conservation and Germplasm Innovation in Mountainous Region (Ministry of Education), Guizhou University, Guiyang 550025, China

**Keywords:** *Pinus massoniana*, drought, coexpression, hormone, peroxisome

## Abstract

*Pinus massoniana* Lamb. is an important coniferous tree species in ecological environment construction and sustainable forestry development. The function of gene gradual change and coexpression modules of needle and root parts of *P. massoniana* under continuous drought stress is unclear. The physiological and transcriptional expression profiles of *P. massoniana* seedlings from 1a half-sibling progeny during drought stress were measured and analyzed. As a result, under continuous drought conditions, needle peroxidase (POD) activity and proline content continued to increase. The malondialdehyde (MDA) content in roots continuously increased, and the root activity continuously decreased. The needles of *P. massoniana* seedlings may respond to drought mainly through regulating abscisic acid (ABA) and jasmonic acid (JA) hormone-related pathways. Roots may provide plant growth through fatty acid β-oxidative decomposition, and peroxisomes may contribute to the production of ROS, resulting in the upregulation of the antioxidant defense system. *P. massoniana* roots and needles may implement the same antioxidant mechanism through the glutathione metabolic pathway. This study provides basic data for identifying the drought response mechanisms of the needles and roots of *P. massoniana*.

## 1. Introduction

*Pinus massoniana* Lamb. (Fam.: *Pinus*; Gen.: *Pinus*) is widely distributed in China and plays a pivotal role in ecological environment construction and sustainable forestry production. Drought is the most widespread stress factor affecting plant productivity [1]; tree radial growth is mainly limited by dry conditions from May to October, with drought-driven tree mortality [2]. Climate predictions have forecast that hot and dry events will become more frequent in the coming decades [3,4]. Molecular responses to drought stress have been extensively studied in broadleaf species, but studies of needle-leaf species have been limited [5]. Studying the response of needle-leaf plants under drought plays an important role in screening high-quality drought-resistant germplasm resources and revealing the mechanism of drought resistance.

In recent years, the study of drought in *P. massoniana* has focused on the photosynthetic characteristics, plasma membrane structure, osmotic adjustment, endogenous enzyme activity, and endogenous hormone content of seedlings under drought stress; most studies tend to be basic theoretical research [6]. Drought significantly reduces the photosynthetic rate, increment of stem basal diameter and plant height, and biomass accumulation [7]. *P. massoniana* enhances its drought resistance by altering the ratio of monoterpenes and sesquiterpenes: the relative contents of monoterpenes decreases with the intensity of drought stress, and the relative content of sesquiterpenes increases [8,9]. The drought resistance of *P. massoniana* is controlled by genetic factors; the differences in drought resistance between different families may be related to the genetic differences formed by long-term domestication in different geographical environments [10]. The response to stress is through the regulation of numerous complex and interrelated metabolic networks. The transcriptome is essential for interpreting the functional elements of the genome, revealing the molecular components of tissues and cells and providing an understanding of development [11]. The underground part mainly shows the growth strategy of “increasing” the root system of *P. massoniana*, which grows in an obvious manner. Transcription factors (TFs) play an important role in the adaptation of *P. massoniana* needles to drought stress [12,13]. However, there is no research on the continuous gradual change of genes and the role and function of needles and roots under continuous stress conditions. In this study, a high-throughput sequencing method is used to identify the gene expression differences between the needles and roots of a half-sibling progeny of *P. massoniana* under continuous drought. This study provides basic data for screening drought resistance factors and understanding drought resistance mechanisms.

## 2. Results

### 2.1. Physiological and Biochemical Analyses

**Figure 1:**
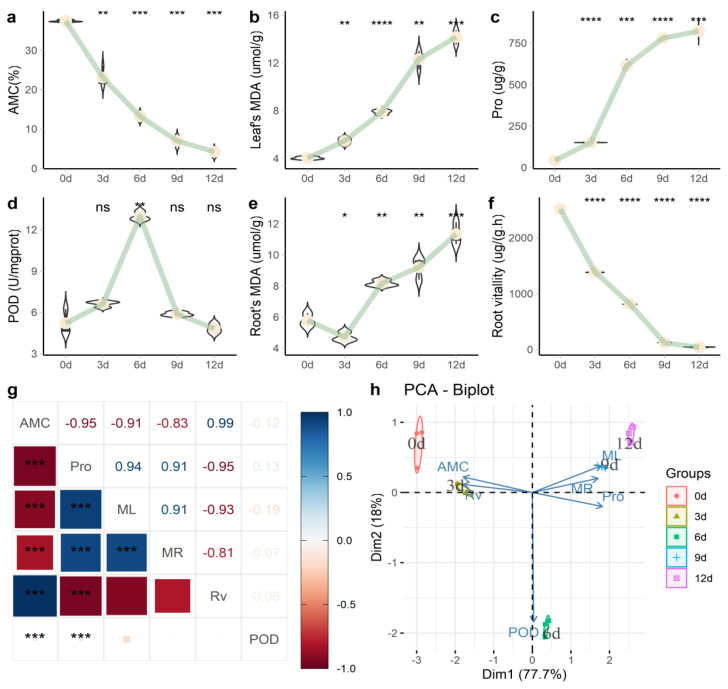
Physiological changes of *P. massoniana* under persistent drought stress. (**a**) Absolute water content in soil (AMC); (**b**) malondialdehyde (MDA) content in needles; (**c**) needle proline (Pro) content; (**d**) needle peroxidase (POD) activity; (**e**) MDA content in roots; (**f**) root activity; (**g**) correlation analysis of various biochemical indicators; (**h**) PCA analysis. Note: In Figure 1a–f, we used the “0 d” group as a reference to compare the mean and added *p*-value and significance markers (“*” indicates that there is a difference compared with the “0 d” group; * *p* < 0.05, ** *p* < 0.01, *** *p* < 0.001, **** *p* ≤ 0.0001 and “ns” means no difference). In Figure 1g, the number represents the magnitude of the correlation, and the color represents the positive and negative correlations.

Through physiological measurements of the process of drought stress (Figure 1), soil absolute moisture content (AMC) gradually decreased (Figure 1a); the plant needles’ malondialdehyde (MDA) (ML) activity (Figure 1b) and the content of needle proline (Pro) (Figure 1c) also continued to rise. Needle peroxidase (POD) activity (Figure 1d) reached its peak in 6 d, after which the needle content continued to decline. Root MDA (MR) activity (Figure 1e) and root activity (RV) (Figure 1f) continued to increase over time. Pro and ML showed a significant positive correlation (*p* = 0.94), while RV and Pro showed a significant negative correlation (*p* = −0.95) (Figure 1g). Principal component analysis (PCA) analysis (Figure 1h) found that the difference between the 6-d group and other groups was mainly reflected in POD activity.

### 2.2. Quality Control, Annotation, and Correlation Analysis

From the quality control statistics of the raw data, it was found that the base efficiency of each sample was high, the base content distribution was uniform, the reads were distributed relatively uniformly, and the Q30 ratio in all samples exceeded 90%, indicating that the sequencing quality was good and could be used for later analysis. After being assembled, 126,925 unigenes were obtained, with an average length of 858.5 bp. Functional annotation revealed that the NR annotation rate was 63.63%, and the Swiss-Prot annotation rate was 36.54%; the Pfam annotation rate was the lowest (0.08%). The raw data of transcriptome are stored in the NCBI/SRA database (BioProject accession No.: PRJNA693351).

**Figure 2:**
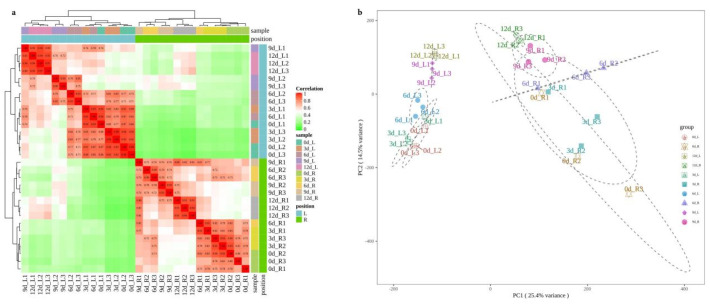
Correlation between samples and PCA analysis. (**a**) Correlation heatmap between samples (the correlation value is greater than 0.7; the correlation method selects person correlation); (**b**) principal component analysis (ellipse shows 95% confidence interval).

The correlation heatmap between samples (Figure 2a) and PCA of unigene expression levels between samples (Figure 2b) showed that the reproducibility between sample groups was good, and the expression patterns were mainly divided into two highly correlated categories.

**Figure 3:**
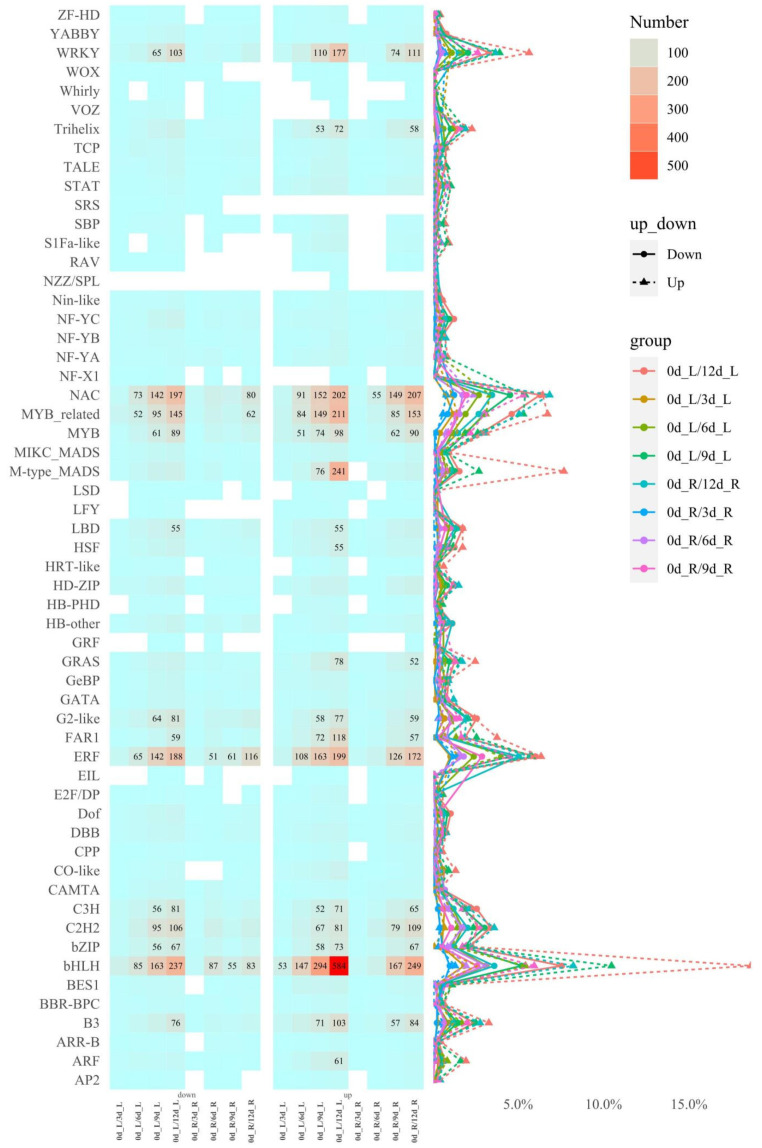
Heatmap and dotted line diagram of differentially expressed genes (DEGs) of transcription factors (TFs) in different periods. Note: The value in the heatmap is the number of DEGs corresponding to the comparison combination, and the value of DEG >50 is displayed. The left heatmap is downregulated, and the right heatmap is upregulated; the percentage value represents the ratio of up/downregulation of TFs in different combinations.

After screening for the differentially expressed genes (DEGs), with the increase in time, the up/downregulated genes of the needle-group-related groups gradually increased (Figure S1). In the 0d_R/6d_R group, the upregulated DEGs reached 4491 (64.67%); 5279 DEGs were specifically differentially expressed in 0d_L/12d_L (Figure S2a), and there were 2227 DEGs between needles and roots in different periods (Figure S2b). In the 0d_R/6d_R group, the upregulated DEGs reached 4491 (64.67%), and there were 3752 DEGs with specific differential expression related to tissue specificity (Figure S2c). Among these different groups of DEGs, bHLH-, NAC-, and MYB-related TFs had the highest proportion (Figure 3). KEGG pathway enrichment analysis shows that in the different teams of needles, plant hormone signal transduction (ko04075) was always enriched, occupying an important role (Figure S3).

### 2.3. Short Time-Series Expression Miner Analysis

**Figure 4:**
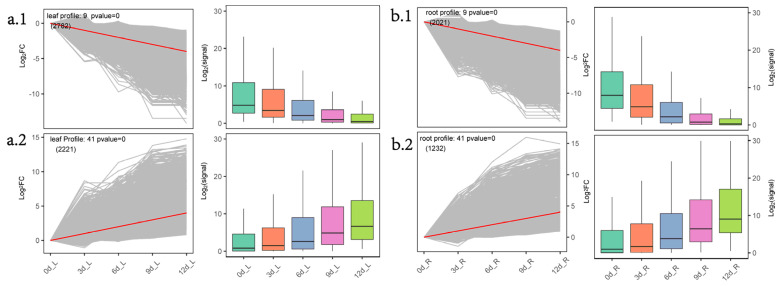
Cluster analysis of gene expression trends during drought stress in *P. massoniana.* (**a.1**) and (**a.2**): Two STEM significance modules (needle Profile9 and needle Profile41) of the needles during drought stress; (**b.1**) and (**b.2**): Two short time-series expression miner (STEM) significance modules (root Profile9 and root Profile41) of the roots during drought stress. Note: We used STEM software to perform trend clustering analysis and divided the trends into 5 stages according to the time before and after input and normalized the union of all the combined DEGs from the five stages. The left side of each subfigure is the significant module selected in the STEM analysis; the right side is the box plot of the corresponding gene expression after taking the logarithm.

The results of short time-series expression miner (STEM) analysis showed that there were 13 significant modules in the needle group and 10 significant modules in the root group. Both the Profile9 module in the needles (Figure 4a.1) and the Profile9 module in the roots (Figure 4b.1) showed the same gene expression trend (0, −1, −2, −3, −4) with prolonged drought—the expression of modular genes continued to decline. KEGG enrichment illustrated that photosynthesis (ko00195), photosynthesis-antenna proteins (ko00196), porphyrin and chlorophyll metabolism (ko00860), and other metabolic pathways were enriched in needle Profile9. Phenylpropanoid biosynthesis (ko00940), ubiquinone and other terpenoids–quinone biosynthesis (ko00130), and other metabolic pathways were enriched in root Profile9. In the ko00940 pathway, most (25/31) genes belong to peroxidase (K00430). In the ko00130 pathway, 10/13 genes belong to tocopherol O-methyltransferase (K05928: E2.1.1.95).

The Profile41 module in needles (Figure 4a.2) and the Profile41 module in roots (Figure 4b.2) both showed 0, 1, 2, 3, 4 gene expression trends. With prolonged drought, the expression of the module gene continued to rise. KEGG enrichment analysis revealed that alpha-linolenic acid metabolism (ko00592), plant hormone signal transduction (ko04075), arachidonic acid metabolism (ko00590), and other metabolic pathways were enriched in the needle Profile41 module. In the ko00592 pathway, 8 genes, including ADH1 (alcohol dehydrogenase 1) and 6 OPRs (12-oxophytodienoate reductase), were enriched. In the ko04075 pathway, related genes, such as PP2C in the ABA pathway and MYC2 in the JA pathway, were enriched. In the ko00590 pathway, glutathione peroxidase (gpx: K00432) was mainly (11/14) enriched. KEGG enrichment analysis revealed that fatty acid degradation (ko00071), alpha-linolenic acid metabolism (ko00592), galactose metabolism (ko00052), fatty acid metabolism (ko01212), and other metabolic pathways were enriched in the root Profie41 module. In the ko00071, ko00592, and ko01212 pathways, genes such as MFP2 (peroxisomal fatty acid beta-oxidation multifunctional protein), ADH1, ADH7A1 (aldehyde dehydrogenase family 7 member A1), acyl-coenzyme A oxidase were enriched. In the ko00052 pathway, galactinol synthase (GolS) was enriched.

### 2.4. Weighted Gene Coexpression Network Analysis (WGCNA) Analysis

By analyzing the needle and root coexpression module of *P. massoniana* during drought, the coexpressed gene set collection was determined, and the analysis revealed several main subexpression profiles with similar expressions, called coexpression modules [14]. We generated a consensus network to identify conserved modules that are needle set-specific and root set-specific in *P. massoniana*.

**Figure 5:**
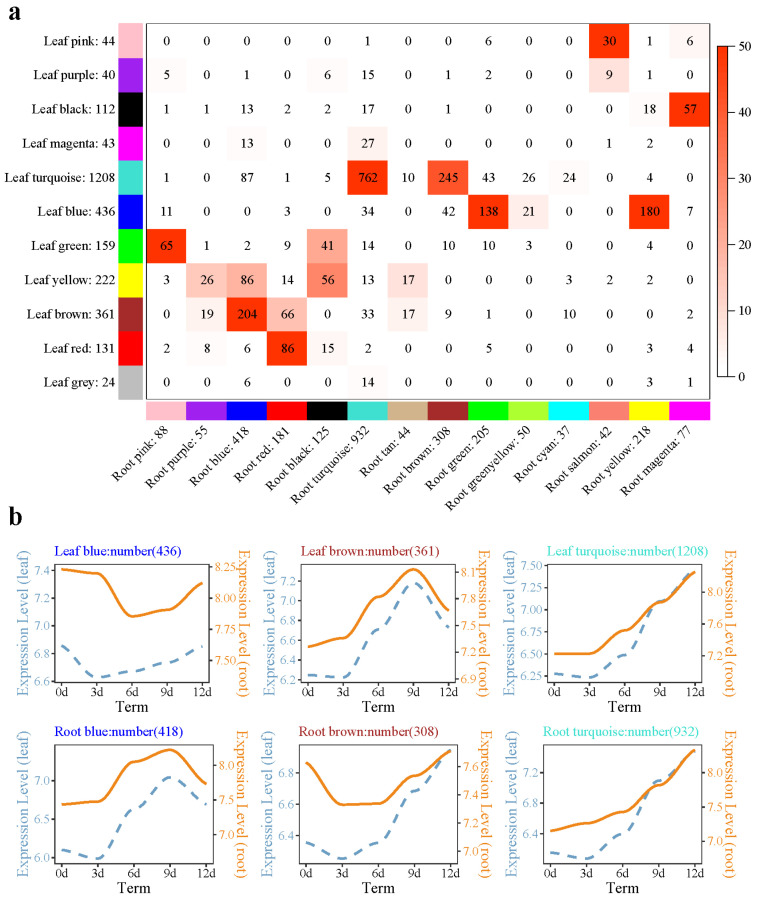
Root and needle coexpression network analysis of the *P. massoniana* drought process. (**a**) Overlap between needle set-specific and root set-specific modules in *P. massoniana*; (**b**) different module gene expression patterns in needles and roots. Note: In subfigure (**a**), each row in the table corresponds to one needle set-specific module, and each column corresponds to one root set-specific module. The number in the labeled heatmap indicates the genes counted in the intersection between two parts of *P. massoniana*. Coloring of the table indicates significant overlap, evaluated using Fisher’s exact test. In subfigure (**b**), the left side of the double-axis represents the needles, the right side represents the roots, the text includes the number of modules and module names, and the gene trends in the module are processed by taking the logarithm of the gene expression in the module, fitted in a linear simulation.

A total of 11 modules (24 to 1208 genes) were identified in the needle module, and a total of 14 modules (37 to 932 genes) were identified in the root module (Figure 5a). In the needle module, the genes of the black and magenta submodules showed a downward trend with drought time. GO enrichment showed that flavonoid biosynthesis (GO: 0009813), the flavonoid metabolism process (GO: 0009812), and other biological processes were enriched in the megenta submodule. The genes of the turquoise submodule in needles showed an increasing trend with prolonged drought time (Figure 5b). KEGG pathway enrichment analysis found that glutathione metabolism (ko00480), MAPK signaling pathway-plant (ko04016), flavonoid biosynthesis (ko00941), and other pathways were enriched. In the root module, the turquoise submodule and the needle submodule shared the most genes, up to 762. GO enrichment of these 762 genes showed that phospholipid–hydroperoxideglutathione peroxidase activity, glutathione peroxidase activity (glutathione peroxidase activity), and other molecular functions were enriched.

## 3. Discussion

Large-scale biogeographical shifts in forest tree distributions are predicted in response to altered precipitation and temperature regimes associated with climate change [15]. Seed sources with higher plasticity perform better in core habitat conditions [16]. Paying attention to the response of forest species to drought will help us use molecular markers and field experiments to screen for drought-resistant core germplasm resources, which are necessary to improve forest productivity under the conditions of climate change. Transcription differences between different tissue parts/organs are essential for a comprehensive understanding of the stress response of the entire plant. By measuring the physiological indexes of needles and roots during drought of the half-sibling progeny of *P. massoniana*, the gene expression profiles during the drought process were constructed. This study provides further insights into the process of drought response and tolerance in *P. massoniana*.

In response to increased H_2_O_2_ levels due to stress, plants have evolved different enzymatic and nonenzymatic mechanisms, including free radical scavengers such as superoxide dismutase, catalase, and peroxidase, as well as ascorbic acid-glutathione circulating enzymes [17,18]. During drought, total NSC (TNSC, soluble sugars plus starch) values decrease, particularly in leaves of *P. sylvestris* L. [19]. MDA and Pro, SOD activity, root shoot ratio, and root mass ratio increased significantly during drought treatment in *P. massoniana* [20]. Under drought conditions, MDA in needles (Figure 1b) and the content of proline continued to rise (Figure 1c), the MDA in roots (Figure 1e) continued to rise, and root vitality (Figure 1f) continued to decline.

As a sun plant, *P. massoniana* has higher light compensation and saturation points and a stronger ability to adapt to strong light. Under moderate (field-water-holding capacity 35%–45%) and severe (20%–30%) drought stress, net photosynthetic rate and transpiration rate decreased significantly [21]. The reduction of photosynthesis and chlorophyll contents under drought stress is the most direct manifestation of plants under drought stress. KEGG pathway enrichment analysis showed that plant hormone signal transduction (ko04075) was always enriched in the different teams of needles (Figure S3). STEM analysis found that photosynthesis (ko00195), photosynthesis-antenna proteins (ko00196), porphyrin and chlorophyll metabolism (ko00860), and other metabolic pathways were enriched in the needle Profile9 module (Figure 4a.1). The modules’ inner genes showed the expression trend 0, −1, −2, −3, −4. ADH1, as one of the marker genes for drought or ABA response, is induced by high ABA levels in *Arabidopsis* under drought stress [22,23]. Overexpression of *AtADH1* increases the transcription levels of multiple stress-related genes and the accumulation of soluble sugars and callose deposition and enhances the abiotic and biological stress resistance of *Arabidopsis* [24]. During drought stress induced by PEG in *Arabidopsis thaliana*, *ADH1* and two other *ADH* genes responded to drought in needles and roots together [25]. Under drought stress, 24 *ADH1* were upregulated in the needle Profile41 module, and 12 *ADH1* were upregulated in the needle Profile41 module. In cell signaling, protein kinases (PKs) and phosphatases (PPs) are key enzymes in the regulation mechanism of protein reversible phosphorylation [26]. Plant PP2Cs participate in various signal cascades, such as ABA and salicylic acid (SA)–ABA crosstalk [27]. OPR is involved in the biosynthesis of JA. The key process in the synthetic pathway is the conversion of cis-12-oxo-docosadienoic acid (OPDA) to 12-oxo-phytoenoic acid (OPC-8:0), catalyzed by OPR; the reaction takes place on the peroxisomes [28,29,30]. The function of OPR may be conserved in higher plants, and rice *OsOPR7* can make up for the phenotype of *Arabidopsis opr3* mutants [31]. In the Profile41 module in needles (Figure 4a.2), 8 *ADH1*, 6 *OPRs*, and *PP2C* in the ABA pathway, MYC2 in the JA pathway, and other related genes were enriched, indicating that the needles of *P. massoniana* may mainly regulate ABA and JA-hormone-synthesis-related pathways in response to drought.

The major ROS regulatory enzyme systems in plant peroxisomes include catalase, all ascorbic acid-glutathione cycle components, and superoxide dismutase (SOD) [32]. Peroxisomes contribute to several metabolic processes, such as β-oxidation of fatty acids, biosynthesis of ether phospholipids, and metabolism of ROS. β-oxidation of fatty acids and detoxification of ROS are generally accepted as being fundamental functions of peroxisomes [33,34]. β-oxidation consists of four enzymatic steps: ACX oxidation, MFP2 hydration and dehydration, and 3-ketoacyl-CoA thiolase (KAT) thiolysis. β-oxidation degrades fatty acids only in the peroxisomes in plants and their derivatives [35]. Peroxisomal abundance can be used as a cellular sensor for drought and heat stress responses; severe drought stress induces peroxisomal proliferation and affects the size of the peroxisomes [36,37]. GOLS is a key rate-limiting enzyme for raffinose synthesis [38]. Overexpression of *AtGOLS2* in rice significantly improved the drought tolerance of transgenic rice; at the same time, the yield of transgenic rice lines under drought conditions was significantly higher than that of the control [39]. In the root Profie41 module (Figure 4b.2), ko00071, ko00592, and ko01212 pathways were enriched, and genes such as *MFP2*, *ADH1*, *ADH7A1,* and *ACOX* were enriched. In the ko00052 pathway, genes such as GOLS were enriched, and related differential genes presented gene expression trends of (0,1,2,3,4). KEGG pathway enrichment analysis found that phenylpropanoid biosynthesis (ko00940) was enriched in the root Profile9 module (Figure 4b.1); most (25/31) of the genes belong to peroxidase (K00430). β-oxidation is believed to enhance the production of stress-induced ROS, which adversely affects plant survival under adverse environmental conditions [40]. Under drought stress, plants tend to increase ROS levels, which, in turn, leads to an upregulation of the antioxidant defense system [41]. This shows that the declining peroxidase-related genes may act as inhibitor agents, but the related regulatory mechanisms are currently unclear. On the other hand, fatty acids are catabolized by β-oxidation to acetyl-CoA, producing sugar, glyoxylic acid, and sugar for seedling growth and development without photosynthesis xenobiosis [42]. This shows that under continuous drought conditions of *P. massoniana*, the roots may provide plant growth through fatty acid β-oxidative decomposition, and *P. massoniana* peroxisomes may contribute to the production of ROS, resulting in the upregulation of the antioxidant defense system.

*P. pinaster* root, stem, mature, and aerial organs, under nontarget metabolomics analysis, were found to have a large number of flavonoids, detected in aerial organs, mainly in root glutathione pathway induction. It is believed that aerial organs and roots activate different antioxidant mechanisms [43]. Transcriptome clustering analysis of *P. halepensis* found that ROS were cleared by an ascorbic acid–glutathione cycle, fatty acid and cell wall biosynthesis, stomatal activity, and biosynthesis of flavonoids and terpenoids [5]. The drought-tolerant seed sources of *P. halepensis* showed increased levels of glutathione, methionine, and cysteine [44]. The *P. massoniana* glutathione peroxidase gene, *PmGPX6*, is highly expressed in the roots. Overexpression of *PmGPX6* in Arabidopsis and wild-type plants have little difference in phenotype and root length under normal water conditions, but under drought stress, in transgenic plants, the root system is longer [45]. Phospholipid hydroperoxide glutathione peroxidase, peroxidase, and other activities are related to reducing the accumulation of reactive oxygen in stress-induced environments [46]. The coexpression module of phospholipid–hydroperoxideglutathione peroxidase activity, glutathione peroxidase activity, and other molecular functions in the roots and needles of *P. massoniana* were enriched. It shows that the glutathione metabolic pathway between the roots and needles of *P. massoniana* may implement the same mechanism of activating antioxidants.

## 4. Materials and Methods

### 4.1. Acquisition of Test Materials

The seeds of *P. massoniana* were obtained from the excellent half-sibling progeny of Ma’anshan Seed Garden (26°16′ N and 107°31′ E), Duyun, Guizhou Province, China. These seeds were placed in a greenhouse to cultivate the seeds into annual seedlings. The soil type in the pots was humus:yellow soil (1:3). Natural drought stress was simulated, and plants were normally irrigated for 3 days before drought stress. We started recording after stopping irrigation. We selected three independent plants for destructive sampling, every three days. The sampling time node was 0 (0 d), 3 (3 d), 6 (6 d), 9 (9 d), and 12 days (12 d). Sampling was conducted between 8:00–9:00 in the morning. The root tips (R) of *P. massoniana* were taken; mature needles (L) (removal of needle sheath tissue) in the top meristem were taken; phenotypic changes were also recorded. The labeled samples were immediately washed with double-distilled water and placed in liquid nitrogen for quick freezing.

### 4.2. Determination of Physiological Indicators

We used the methods described in The Principle and Technology of Plant Physiology and Biochemistry Experiments book [47] to determine the following indicators. The aluminum box soil drying method was used to determine the soil absolute moisture content. Root activity (Rv) was determined by the triphenyl tetrazolium chloride (TTC) method [48]. The level of malondialdehyde (MDA) content in the needles (Ml) and roots (Mr) was determined by the thiobarbituric acid (TBA) method [49]. The needles’ peroxidase (POD) activity was determined using guaiacol and hydrogen peroxide as substrates of the reaction, measured at 470 nm [50]. The needles’ proline (Pro) content was measured at 520 nm, according to the method described by Bates et al. [51]. The experiment included three biological and technical replicates. The statistics and analysis of the physiologically related data were performed using R software (https://www.r-project.org/) (accessed on 1 March 2021). Correlation analysis was performed using the ggcorrplot2 package (https://github.com/caijun/ggcorrplot2) (accessed on 1 March 2021). Principal component analysis (PCA) was performed using the FactoMineR software package [52].

### 4.3. Establishment of Transcriptome Library

The total mRNA from the above samples was isolated according to the manufacturer’s instructions for the Trizol reagent (Invitrogen, Carlsbad, CA, USA). Nanodrop 2000 (Thermo Fisher Scientific, Waltham, MA, USA) was used to determine the concentration and purity of the mRNA. RNA integrity was assessed by agarose gel electrophoresis, while its integrity number (RIN) value was measured using an Agilent 2100 (Agilent Technologies, Santa Clara, California, USA). The mRNA extraction quality and concentration of all samples passed inspection (A260/280 = 2.0~2.2; A260/230 = 1.8~2.2; 28S/18S = 1.4~2.7; Rin ≥ 8.0); the mRNA was enriched with Oligo (dT) magnetic beads. Furthermore, fragmentation buffer was added to the mRNA and cut into short fragments. Using the mRNA as templates, cDNA was reverse-transcribed using six-base random primers. The double-stranded cDNA samples were purified, end-repaired, added with poly(A) tails, and then ligated to the sequencing adapters to create cDNA libraries. After the libraries passed a quality test, a total of thirty paired-end libraries were used for transcriptome sequencing with an Illumina HiSeq X Ten machine.

### 4.4. Data Processing and Analysis

Trimmomatic software [53] was used to perform quality control on the raw data, and raw reads with adapters or poor-quality sequences were removed. Assembly of the reads was performed using Trinity [54] with default parameters. We used CD-HIT [55] software clustering to reduce redundancy to obtain unigenes. Unigenes were blast to NR, KOG (eukaryotic ortholog groups), GO (Gene Ontology, http//www.geneontology.org; accessed on 1 March 2021), Swiss-Prot, eggNOG, and KEGG (Kyoto Encyclopedia of Genes and Genomes, http://www.genome.jp/kegg; accessed on 1 March 2021) databases. HMMER3 [56] was used to analyze functionality by comparison of unigenes to the Pfam database. Bowtie2 [57] was used to obtain the number of reads aligned to unigenes, the expression of unigene fragments per kilobase of transcript per million mapped reads (FPKM) was calculated using eXpress [58]. DEGs between terms were also identified based on the number counts of unigenes using DESeq [59]. The differentially expressed genes (DEGs) screening threshold was set to p-value <0.05 and |foldchange| >2. The clusterProfiler package [60] was used to perform the GO and KEGG enrichment of DEGs. Short time-series expression miner (STEM) software [61] was used to perform the trend clustering analysis. WGCNA [62] was used to determine the coexpressed gene modules. Median absolute deviation (MAD) was used to screen the common parts of the genes in the top 5000 genes of the needles and roots (2780).

## 5. Conclusions

This study investigates the continuous gradual change process of *P. massoniana* genes under continuous drought stress. Under continuous drought, POD activity and proline content of needles continue to increase; root activity continuously decreases. The needles of *P. massoniana* seedlings may respond to drought mainly through regulating ABA and JA hormone-related pathways. Roots may provide plant growth through fatty acid β-oxidative decomposition, and peroxisomes may contribute to the production of ROS, resulting in the upregulation of the antioxidant defense system. *P. massoniana* roots and needles may implement the same antioxidant mechanism through the glutathione metabolic pathway.

## Data Availability

The raw data of transcriptome have been stored in the NCBI/SRA database (BioProject accession No.: PRJNA693351).

## Supplementary Materials

The following are available online at https://www.mdpi.com/article/10.3390/plants10040769/s1. Figure S1. Histogram diagram of DEGs between needles and roots in different periods. Figure S2. Venn diagrams of DEGs of *P. massoniana* in different periods. (**a**) Venn diagram of DEGs at different stages of needles in *P. massoniana*; (**b**) Venn diagram of DEGs between needles and roots at different stages; (**c**) Venn diagram of DEGs at different stages of roots in *P. massoniana.* Figure S3. KEGG enrichment of DEGs in needles and roots in different periods. Note: Each combination selects 10 groups, from small to large, according to *p*-values.
