# Supplementary figures and images for: Transcriptome Analysis of Needle and Root of Pinus Massoniana in Response to Continuous Drought Stress
Source: Plants (Basel). 2021 Apr 14;10(4):769. doi: 10.3390/plants10040769 (PMC8070838; doi:10.3390/plants10040769)

**a**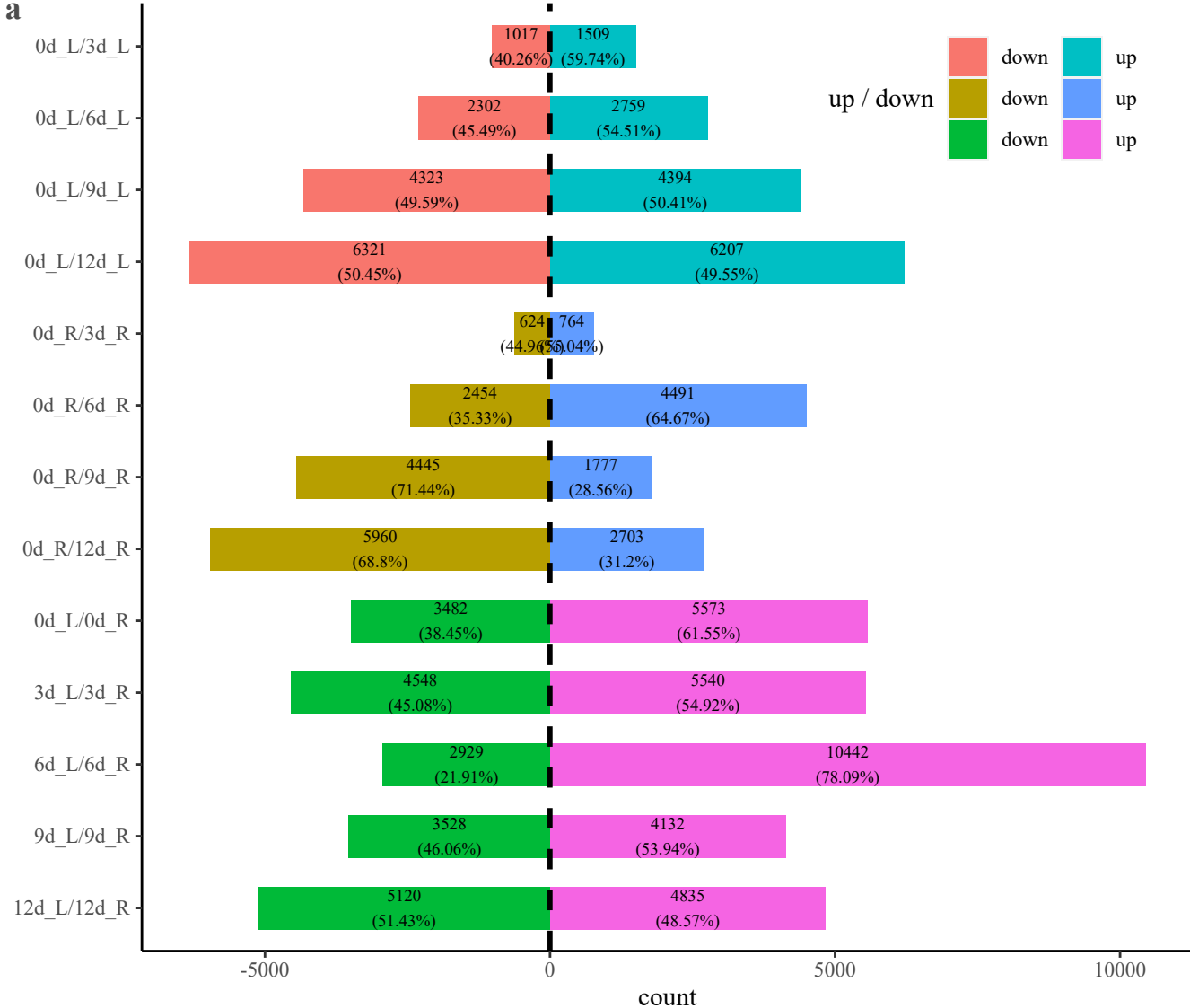

Supplement: Supplementary file 1 [file plants-10-00769-s001.zip › Supplementary Figure/Supplementary Figure S1.pdf]

**a**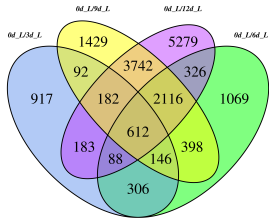**b**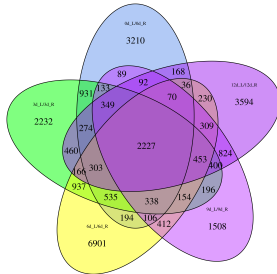**c**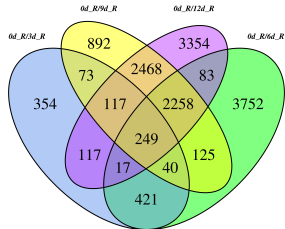

Supplement: Supplementary file 1 [file plants-10-00769-s001.zip › Supplementary Figure/Supplementary Figure S2.pdf]

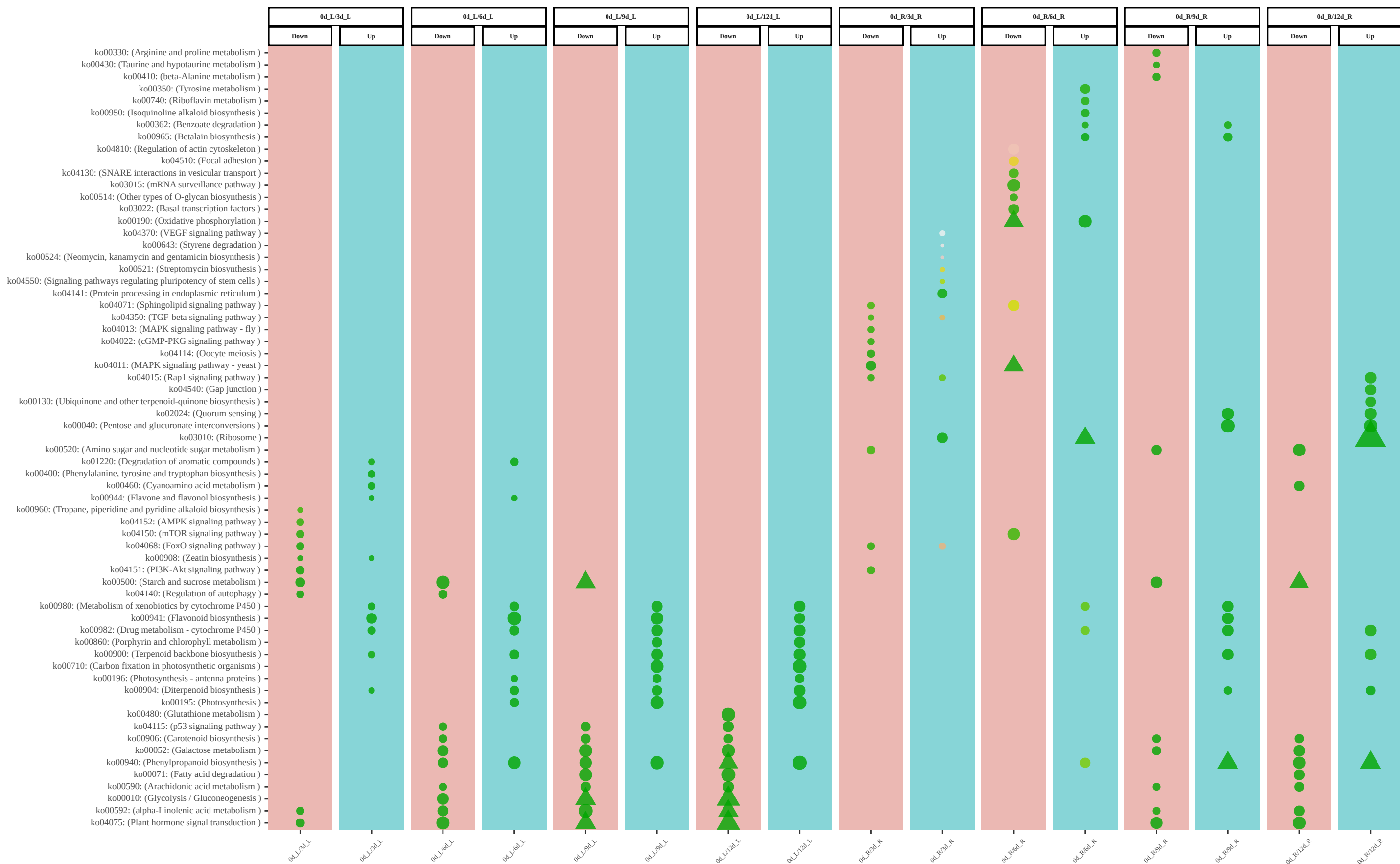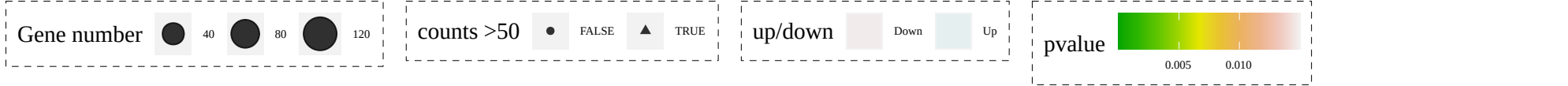

Supplement: Supplementary file 1 [file plants-10-00769-s001.zip › Supplementary Figure/Supplementary Figure S3.pdf]
